# Risk prediction of combined fibrinogen degradation products, thrombomodulin, and maximum amplitude for assessing association with venous thromboembolism in patients with post-traumatic fractures

**DOI:** 10.1371/journal.pone.0346726

**Published:** 2026-04-23

**Authors:** Shaoli Huang, Xiaoyan Wang, Hongwu Xin, Xihe Zhang, Wenying Luo

**Affiliations:** 1 Medical laboratory Center, Affiliated Hospital of Guangdong Medical University, Zhanjiang, Guangdong, China; 2 First Clinical College, Guangdong Medical University, Zhanjiang, Guangdong, China; 3 Clinical laboratory, Lianjiang People’s Hospital, Lianjiang, Guangdong, China; 4 Doctoral Scientific Research Center, Lianjiang People’s Hospital, Liangjiang, Guangdong, China; 5 Guangdong Medical University Affiliated Lianjiang People’s Hospital, Liangjiang, Guangdong, China; 6 Key Laboratory of Biomedical Engineering Technology at Universities of Shandong Province, and Special Laboratory of Medical Biotechnology and Functional Materials of Shandong Province, School of Basic Medical Sciences, Qilu Medical University, Zhoucun District, Zibo, Shandong, China; Tekirdag Namik Kemal University: Tekirdag Namik Kemal Universitesi, TÜRKIYE

## Abstract

**Background:**

Venous thromboembolism (VTE) is a globally prevalent severe disease associated with high morbidity and mortality. Currently, thrombomodulin (TM), fibrinogen degradation product (FDP), thromboelastography have been the subject of several research pertaining to VTE; However, the combined diagnostic efficacy of these tests for VTE remains unclear. Therefore, we proposed to investigate the diagnostic efficacy of TM, FDP, thrombelastography in predicting VTE.

**Methods:**

The patients with traumatic fracture included in the study were divided into a VTE group (n = 44) and a control group (n = 56) based on imaging diagnosis. Spearman correlation was employed to analyze the relationship between coagulation-related markers and thromboelastography indices. Variables were analyzed using multifactorial logistic stepwise regression. Statistically significant indicators were included in the receiver operating characteristic curve to evaluate their diagnostic efficacy for VTE.

**Results:**

The VTE group showed significantly higher levels of multiple coagulation-related parameters and thromboelastography indices compared to the control group. Specifically, D-dimer levels were 8.87 (4.77, 15.07) mg/L in the VTE group versus 2.36 (1.07, 5.73) mg/L in the control group (*P* < 0.001), and FDP levels were 36.45 (11.34, 73.75) μg/mL versus 7.96 (4.57, 12.73) μg/mL (*P* < 0.001). TM levels were also elevated in the VTE group at 11.74 (9.26, 13.27) TU/mL compared to 8.60 (7.20, 11.60) TU/mL in controls (*P* = 0.001). Among thromboelastography parameters, maximum amplitude (MA) was 72.06 ± 7.61 mm in the VTE group versus 67.03 ± 7.21 mm in controls (*P* = 0.001), and clot intensity (G) was 13,259.75 (9,346.48, 18,545.83) d/sc versus 9,659.70 (8,009.33, 13,480.40) d/sc (*P* = 0.004). Conversely, the blood clot formation rate was lower in the VTE group (1.25 [0.83, 1.40] vs. 1.30 [1.10, 1.58], *P* = 0.037). Linear correlation analysis revealed significant positive associations between platelet counts and both MA (r = 0.612, *P* < 0.001) and G (r = 0.588, *P* < 0.001). Multivariate logistic stepwise regression identified FDP (OR = 1.047, 95% CI: 1.025–1.070, *P* < 0.001), TM (OR = 1.215, 95% CI: 1.033–1.429, *P* = 0.019), and MA (OR = 1.104, 95% CI: 1.026–1.188, *P* = 0.008) as independent risk factors for VTE. ROC curve analysis demonstrated that the combined model of these three markers achieved the highest diagnostic efficacy, with an area under the curve (AUC) of 0.860 (95% CI: 0.789–0.931), sensitivity of 70.5%, and specificity of 85.7%.

**Conclusion:**

Combined testing of FDP, TM, and MA holds clinical significance for clinicians to early predict the risk of VTE in post-traumatic fracture patients and implement preventive measures.

## Introduction

Venous thromboembolism (VTE) is one of the severe medical conditions with morbidity and mortality worldwide [1], comprising deep vein thrombosis (DVT) and pulmonary embolism (PE), it is a chronic disorder that impacts around 10 million people worldwide annually [2]. The high prevalence of VTE not only contributes significantly to inpatient mortality but also presents a formidable challenge to hospital administrators and clinical staff. VTE is a multifaceted condition influenced by both acquired risk factors, such as hormone therapy, trauma, surgical procedures, cancer, or immobilization due to hospitalization [3], and inherited risk factors like factor V Leiden carrier patients [4]. Surgical procedures, injuries, or prolonged immobilization are primary causes of acquired venous thrombosis [5]. Trauma patients are at a significantly increased risk of VTE, with a reported prevalence of up to 30%. VTE is an extremely common complication associated with a higher risk of hospitalization and post-discharge mortality [6–9]. DVT often occurs as a complication following lower limb fracture surgery. However, research indicates that only 10–17% of DVT patients have visible clinical symptoms, including lower limb swelling, local deep pressure pain, and dorsiflexion pain. This leads to a high rate of underdiagnosis and misdiagnosis of lower limb DVT. Therefore, from a clinical perspective, it is crucial for high – risk patients to receive timely diagnosis and treatment [10,11]. Currently, the DVT diagnosis primarily depends on ultrasound and angiographic techniques. Although both methods are diagnostically valuable, ultrasound can not diagnose intraperitoneal venous embolization, while angiography, despite its high invasiveness and cost, offers comprehensive evaluation [12]. Moreover, in asymptomatic patients, the underdiagnosis rate of lower extremity DVT by ultrasound is approximately 50% [13]. This is characterized by a low positivity rate and a high false- negative rate [13]. The D-dimer assay is currently used as an ordinary test of screening for patients with suspected DVT. However, it has a high sensitivity but a low specificity of only 40% to 50% [14–16]. The lack of clear clinical indicators and vague symptoms of venous thrombosis can lead to delayed or even misdiagnoses, significantly affecting the patient’s prognosis [17,18]. In certain cases, even after comprehensive screening, the etiology of thrombosis may remain elusive [19,20]. This means that a subset of patients with venous thrombosis might be overlooked. Consequently, there is an urgent requirement to identify specific and sensitive biomarkers for venous thrombosis.

The vascular endothelium maintains thromboresistance via regulated expression of tissue plasminogen activator and other fibrinolytic components. Fibrinolysis not only breaks down the extracellular matrix but also maintains vascular integrity and regulates cell migration, adhesion, and tissue remodeling [21–23]. Activated by thrombin, fibrinogen is converted into fibrin. Elevated levels of fibrinogen can lead to increased blood viscosity, enhanced platelet aggregation, and accelerated thrombus formation [24]. Multiple studies [25,26] have established a causal venous thromboembolism. In patients with lower limb fractures, platelet, D-dimer, fibrinogen, and fibrin degradation products (FDP) were found to be independent predictors of DVT [27].

When vascular damage occurs, the expression of thrombomodulin (TM) is significantly upregulated [28,29]. Clinical investigations have illustrated the potential of TM as a dynamic biomarker for monitoring DVT development [30]. In the study by Zhou K, 2020 investigating the diagnostic and prognostic value of TM, thrombin-antithrombin (TAT), plasmin-α2-plasminogen inhibitor complex (PIC), and tissue plasminogen activator-inhibitor complex (t-PAIC) in patients with malignant tumors and venous thrombosis revealed that the combination of TM, TAT, PIC, t-PAIC, D-dimer, and FDP was more effective than any single biomarker in detecting venous thrombosis in these patients [31]. For patients with traumatic fractures, the diagnostic validity of thrombotic markers for postoperative DVT has been demonstrated to decline in order (AUC: TM > D-dimer > PIC > t-PAIC > TAT), with TAT showing no statistical significance. After traumatic fracture surgery, patients’ risk of developing DVT may be predicted by combining thrombotic molecular markers with postoperative Caprini ratings [32].

Thromboelastography (TEG) provides a comprehensive view of multiple coagulation processes, including early thrombin production, fibrin synthesis, and fibrinolysis. It operates based on the changes in blood viscosity [33]. With the growing application of TEG in trauma patients, clinicians often observe a hypercoagulable state known as fibrinolysis shutdown (SD), when these patients are admitted to the hospital. Alarmingly, up to 22% of patients die due to this condition [34–37]. Persistent hypercoagulability, on the other hand, is an independent risk factor for trauma-related VTE [38,39]. Moreover, a study by Joshua et al. suggested that the maximum amplitude (MA) of thromboelastography testing upon admission identifies patients with severe orthopedic trauma. Patients with MA values of ≥65 mm and ≥72 mm have a 3.6 and 6.7 – fold higher risk of developing PE and DVT respectively during their hospital stay [40]. For trauma patients, early recognition of VTE resulting from trauma is crucial for reducing mortality and other complications associated with VTE. Therefore, this study integrates the endothelial damage caused by traumatic injury, the fibrin formation due to hemostasis, and TEG, which provides a comprehensive view of the coagulation process. The aim of this study is to identify the most appropriate diagnostic parameter for VTE from the coagulation process and establish a diagnostic model for VTE through logistic regression analysis.

## Materials and methods

### Study population

This study was a retrospective case-control study involving all trauma fractures patients with electronic medical records from the Affiliated Hospital of Guangdong Medical University and Yangjiang People’s Hospital from 01/11/2022 to 01/03/ 2024. The data of this study were accessed for research purposes from 01/01/2025 to 16/01/2025. This study has been approved by the Ethics Committee of the Affiliated Hospital of Guangdong Medical University, and the ethical approval number is PJKT204–258.

#### Inclusion & Exclusion criteria.

**Inclusion Criteria:** Patients diagnosed with traumatic fractures (defined as bone discontinuity due to injury, confirmed by X-ray/CT/MRI); Treated at the same institutions (Affiliated Hospital of Guangdong Medical University and Yangjiang People’s Hospital) during the study period (2022–2024); Age ≥ 18 years; Available coagulation marker such as TAT, TM, PIC, D-dimer, FDP, platelet (PLT), and thromboelastography data within 72 hours of admission and before fracture surgery;

**Exclusion Criteria:** Malignant tumors, blood disorders, or history of venous thrombosis; Receiving hormone therapy or anticoagulant treatment before trauma (Table 1); Severe infection (defined as systemic inflammatory response syndrome, SIRS); Pregnancy.

**Table 1:** Clinical data between VTE and control groups of patients with post-traumatic fractures.

	VTE (n = 44)	Control (n = 56)	*P*-value
Age (years)	66.52 ± 12.70	52.39 ± 21.26	<0.001
The time from the patient’s fracture to the patient’s admission for ultrasound examination(days)	5.50 (2.25, 13.75)	4.0 (2.0, 12.0)	0.663
Gender Males	27 (61.4%)	38 (67.9%)	0.499
Females	17 (38.6%)	18 (32.1%)	
Smoking	3 (6.8%)	6 (10.7%)	0.746
Use of anticoagulant drugs	24 (54.5%)	25 (44.6%)	0.325
Diabetes	16 (36.4%)	15 (26.8%)	0.304
D-dimer (mg/L)	8.87 (4.77, 15.07)	2.36 (1.07, 5.73)	<0.001
FDP (μg/mL)	36.45 (11.34, 73.75)	7.96(4.57,12.73)	<0.001
PLT (10^9^/L)	270.00 (213.00, 370.75)	248.00(194.25, 301.50)	0.093
TAT (ng/mL)	19.44 (11.04, 33.50)	9.70 (5.25, 18.70)	<0.001
TM (TU/mL)	11.74 (9.26, 13.27)	8.60 (7.20, 11.60)	0.001
PIC (μg/mL)	2.13 (1.47, 3.08)	1.09 (0.71, 1.88)	<0.001
R (min)	5.65 (4.56, 7.45)	5.85 (4.60, 7.20)	0.890
K (min)	1.25 (0.83, 1.40)	1.30(1.10, 1.58)	0.037
Angle (deg)	72.50 (69.63, 77.10)	70.15(66.13, 74.00)	0.004
MA (mm)	72.06 ± 7.61	67.03 ± 7.21	0.001
CI	2.25 (0.53, 3.68)	1.25 (0.13, 2.50)	0.031
LY30 (%)	0.30 (0.00, 1.18)	0.65 (0.00, 1.73)	0.220
G (d/sc)	13259.75 (9346.48, 18545.83)	9659.70 (8009.33, 13480.40)	0.004

R: reaction time; K: clot formation time; Angle: blood clot formation rate; MA: maximum amplitude; LY30%: estimated percentage of lysis at 30 minutes; G: clot intensity; CI: coagulation index; FDP: fibrinogen degradation product; PLT: platelet; TAT: thrombin-antithrombin; TM: Thrombomodulin; PIC: plasmin-α2-plasminogen inhibitor complex.

### Grouping and diagnostic methods

Postoperatively, all fracture patients underwent lower limb vascular ultrasound within 1 week for primary VTE screening; patients with abnormal ultrasound findings received further CT/MRI. Based on these instrumental diagnostics, patients were divided into VTE and non-VTE groups [41,42]. Diagnostic criteria for VTE were as follows:

**Ultrasonography:** At least one of the following criteria must be met: non-dilated lumen, solid hypoechoic filling within the lumen, failure of lumen collapse under probe pressure, absence of blood flow filling in the lumen, or absence of blood flow signals [24,43].
**CT imaging:** Focal or multicentric filling defects in the venous lumen (eccentric or irregular), venous dilatation, venous wall enhancement, striated involvement of single or multiple vessels, perivenous fat stranding with collateral opacification, or collateral circulation formation [44,45].
**MRI imaging:** Venous filling defects, collateral circulation formation, reduced vessel lumen diameter, or low-signal linear fibrous segments within the lumen [46].

### Sample size calculation

The sample size was precomputed using an online tool (Comparing Two Means, Select Statistical Services) based on the formula:


$$\text{n}=\text{2}{\left({\text{Z}}_{\alpha /\text{2}}+{\text{Z}}_{\beta }\right)}^{\text{2}}\sigma^{\text{2}}/{\text{d}}^{\text{2}}$$

with parameters: 95% confidence interval (Zα/2 = 1.96), 80% power (Zβ = 0.84), estimated variance (σ2 = 1000), and minimum detectable difference (d = 20). This yielded n = 50 per group. Due to retrospective data limitations, the final sample included 44 VTE cases and 56 controls, with baseline characteristics balanced between groups (Table 1).

### Clinical data collection

Clinical data were retrieved from the electronic health record system. The collected information included patients’ gender, age, smoking history, venous thrombosis history, diabetes history, anticoagulant use, and the time interval between trauma occurrence and VTE diagnosis. Additionally, within 72 hours of VTE diagnosis, coagulation – related markers were collected from the patients. These markers consisted of TAT, TM, PIC, D-dimer, FDP, platelet (PLT), and thromboelastography parameters such as reaction time (R), blood clot formation time (K), blood clot formation rate (Angle), maximum amplitude (MA), estimated percentage of lysis at 30 minutes (LY30%), coagulation index (CI), clot intensity (G), and others.

### The indicators of the thromboelastography

The prothrombin generation time, defined as the time interval from the commencement of blood sample detection to the formation of the first clot, is represented by the R value in the thromboelastography. Hypercoagulability tends to shorten the R value, whereas the absence of coagulation factors or the presence of anticoagulants can prolong it. The K – value serves as an indicator of the clot – formation rate and is closely related to the time of thromboplastin production. Additionally, both the K value and Angle reflect the rate of clot formation, and these parameters are predominantly influenced by the quantity and quality of fibrinogen, prothrombin, and platelets ^46^. The MA reflects the maximum strength of the clot formed by fibrin and platelets through the interaction of GPIIb and IIIa receptors [47]. When two or more of the following criteria are met in comparison to the reference range, the blood is classified as hypercoagulable: a decreased R-value, a decreased K- value, an increased Angle, or an increased MA [46,48].

### Main reagents and materials

The reagent of TM, TAT and PIC was bought from Wanfu Biotechnology Co., Ltd. (Guangzhou, China); the reagent of D-dimer and FDP was bought from DIAGNOSTICA STAGO (Asnières-sur-Seine, France); and the reagent of thromboelastography was bought from Lepu Diagnostics Technology Co., Ltd. (Beijing, China). The blood samples from the same patient were drawn simultaneously, avoiding additional blood draws or timing discrepancies.

Collection and Storage of TM, PIC, and TAT specimens: Centrifuge the specimen at 4000 rpm for 10 minutes to separate plasma for testing. If testing cannot be performed immediately after plasma separation, store at 2−8°C. For storage exceeding 24 hours, store at −20°C. TEG specimen collection and storage: Collect blood from the patient on an empty stomach using a sodium citrate tube. Test the specimen immediately. If immediate testing is not possible, store at room temperature (10−30°C) for no longer than 4 hours. FDP, D-dimer: Centrifuge the sample at 2000−2500 g for 15 minutes. Store at 20 ± 5°C for no longer than 8 hours. If storage exceeds 8 hours, store at −20°C for no longer than one month.

### Statistical analysis

SPSS 26.0 was used to conduct the statistical analysis. The heatmap was made with GraphPad Prism 10. The t-test was utilized to evaluate the variations among the groups for continuous variables that followed a normal distribution. When dealing with categorical variables presented as constituent ratios, the chi – square test was used to compare the differences between two groups. For continuous variables with non-normal distributions (reported as percentiles), the Mann – Whitney U test was employed for comparing two groups. It should be noted that the chi – square test is not related to the concept of “chi - square variance” as inaccurately described before. In cases where each group had no more than five cases, Fisher’s exact test was utilized to compare the two groups. To analyze the correlation between coagulation – related markers and various thromboelastography indicators, Spearman correlation was carried out. Multifactorial logistic stepwise regression analysis was used to assess the risk factors of VTE in post-traumatic fracture patients. The diagnostic effectiveness was evaluated using the receiver operating characteristic (ROC) curve, with the optimal threshold determined by taking the highest value of the Youden index.

## Results

In total, there were 100 cases, including 65 males and 35 females. Among them, 44 cases belong to the VTE group, and 56 cases to the control group. The analysis results revealed that there were no statistical differences between the VTE and control groups regarding the time of VTE diagnosis, gender, smoking status, use of anticoagulants, or the presence of diabetes mellitus (Table 1). This indicates that these baseline conditions were in equilibrium after combining the data. Nevertheless, the age was statistically significant difference in the two groups. Moreover, the levels of D-dimer, FDP, TAT, PIC, TM, Angle, G, MA, and CI in the VTE group were markedly higher than those of the control group, with statistical differences of *P* < 0.05. There were no statistical differences between the two groups of PLT, R, and LY30%, and the detailed results are presented in Table 1.

**Fig 1:**
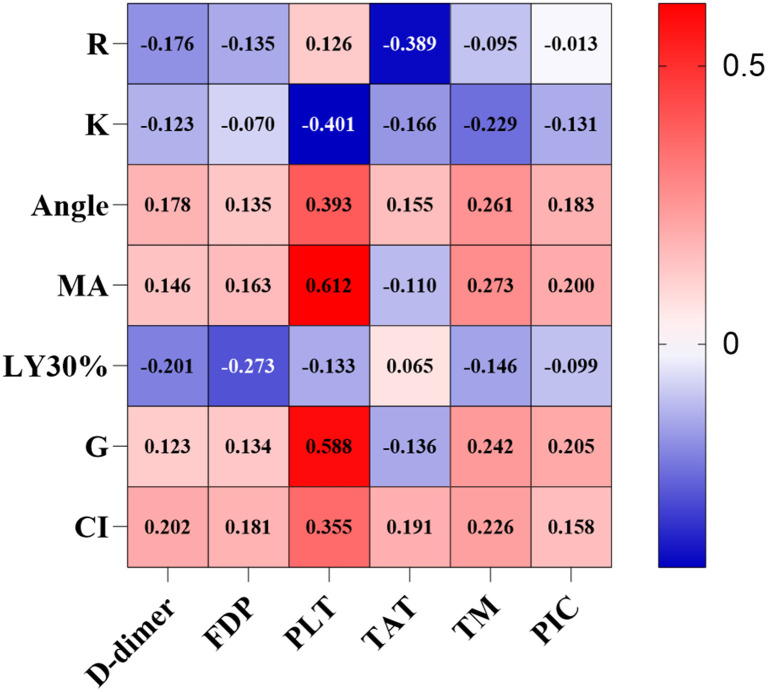
The correlation between thrombus-related indicators and the indicator of Thromboelastography. R: reaction time; K:clot formation time; Angle: blood clot formation rate; MA: maximum amplitude; LY30%: estimated percentage of lysis at 30 minutes; G: clot intensity; CI: coagulation index; FDP:fibrinogen degradation product; PLT: platelet; TAT: thrombin-antithrombin; TM:Thrombomodulin; PIC: plasmin-α2-plasminogen inhibitor complex.

To verify the correlation between the thrombus-related indices and the indicators of the thromboelastography, we used Spearman for correlation analysis due to the non-normal distribution of data, and the analysis results are displayed in Fig 1. The K value, Angle, and CI of thromboelastography exhibit a weak linear correlation with PLT, and the *r*-values are −0.401, 0.393, and 0.355, respectively. Specifically, there is a negative correlation between the K value and PLT, while both CI and Angle had a positive correlation with PLT. MA and G of the thromboelastography exhibited a significantly linearly positive correlation with PLT, with r-values of 0.612 and 0.588, respectively. In addition, a weak negative linear correlation was observed between the R value of the thromboelastography and the thrombin – antithrombin complex (TAT), with an r – value of −0.389. Notably, there was no linear correlation between D-dimer, FDP, TM, and PIC and each indicator of the thromboelastography.

The selected variables were incorporated into the multivariate logistic stepwise regression analysis, and the detailed results are presented in Table 2. The effect of FDP on VTE was statistically significant difference (OR = 1.047, 98% CI 1.025–1.070, *P* < 0.001). The effect of TM on VTE was statistically significant difference (OR = 1.215, 98% CI 1.033–1.429, *P* = 0.019). The effect of MA on VTE was also statistically significant difference (OR = 1.104, 98% CI 1.026–1.188, *P* = 0.008).

**Table 2:** Logistic regression analysis of VTE risk factors in patients with post-traumatic fractures.

variable	B	Standard error	Wald	*P*-value	OR	95%CI
FDP (μg/mL)	0.046	0.011	17.803	<0.001	1.047	1.025 ~ 1.070
TM (TU/mL)	0.195	0.083	5.538	0.019	1.215	1.033 ~ 1.429
MA(mm)	0.099	0.037	7.058	0.008	1.104	1.026 ~ 1.188

FDP: fibrinogen degradation product; TM: thrombomodulin; MA: maximum amplitude; OR: odds ratio; CI: confidence interval.

**Fig 2:**
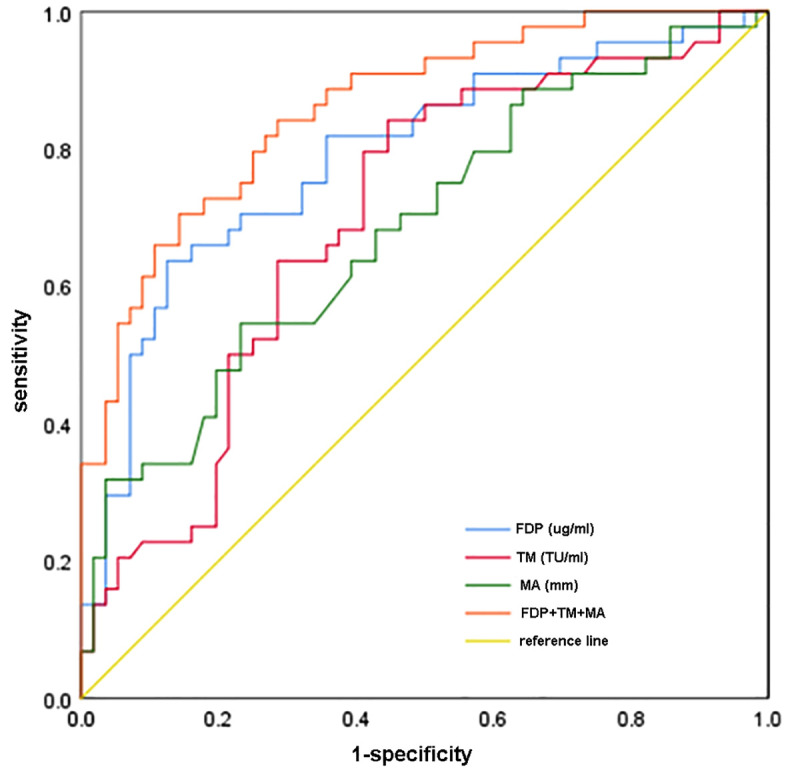
ROC curves were generated to evaluate the diagnostic efficacy of FDP, TM, and MA individually, as well as in combination, for diagnosing VTE in post-traumatic fracture patients. ROC: receiver operating characteristic; MA: maximum amplitude; FDP:fibrinogen degradation product; TM:Thrombomodulin; VTE: venous thromboembolism.

To assess the correlation between thrombus indicators and VTE, we conducted a multivariate analysis and the ROC curve in Fig 2 and detailed in Table 3. Regarding the diagnostic performance of individual indicators, FDP demonstrated a sensitivity of 63.6% in diagnosing VTE, accompanied by a specificity of 87.5% [95% confidence interval (CI): 0.697–0.881]. TM showed a relatively high sensitivity of 84.1% for VTE diagnosis but a relatively low specificity of 55.4% (95% CI: 0.594–0.802). In the context of thromboelastography, MA had a sensitivity of 54.5% for VTE diagnosis, indicating lower sensitivity compared to some other indicators, with a specificity of 76.8% (95% CI: 0.579–0.790). The combined sensitivity of FDP, TM, and MA for diagnosing VTE is 70.5%, while specificity reaches 85.7% (95% CI: 0.789 ~ 0.931). The area under the ROC curve is 0.860, an AUC of 0.860 reflects a strong discriminative ability of this combination of indicators for identifying VTE.

**Table 3:** Diagnostic efficacy of FDP, TM, and MA separately and the combination of three tests in diagnosing VTE in post-traumatic fracture patients.

Variable	AUC	95% CI	*p*	Sensitivity (%)	Specificity (%)
FDP (μg/mL)	0.789	0.697 ~ 0.881	<0.001	63.6	87.5
TM (TU/mL)	0.698	0.594 ~ 0.802	0.001	84.1	55.4
MA/(mm)	0.684	0.579 ~ 0.790	0.002	54.5	76.8
FDP + TM + MA	0.860	0.789 ~ 0.931	<0.001	70.5	85.7

AUC: area under the curve; FDP: fibrinogen degradation product; TM: thrombomodulin; MA: maximum amplitude; OR: odds ratio; CI: confidence interval.

## Discussion

Venous thromboembolism (VTE) is a common and life-threatening postoperative complication in trauma and fracture patients, yet its clinical diagnosis has long been plagued by “dual dilemmas”. On the one hand, the missed diagnosis rate of asymptomatic lower extremity DVT by ultrasound is as high as 50% [2,49,50], while invasive examinations such as computed tomography venography are difficult to be used as routine screening methods due to their high cost and potential risks. On the other hand, although the traditional D-dimer test has high sensitivity (>90%), its specificity is only 40%−50% [4,50,51], which easily leads to overtesting or misdiagnosis. Therefore, there is an urgent clinical need for a set of biomarker panels that balance sensitivity, specificity and convenient implementation, for the early stratification and precision management of VTE risk in trauma and fracture patients.

By conducting a retrospective analysis of clinical data from 100 patients with traumatic fractures, this study confirmed that the combined detection of FDP, TM, and thromboelastography MA is an effective tool for predicting VTE risk in patients with traumatic fractures. Its practical conclusion is not merely “suspecting VTE based on elevated levels of the three indicators”, but rather, based on solid statistical evidence, it verifies that this combination possesses the dual attributes of “independent risk predictive value + superior diagnostic efficacy”. From the perspective of risk prediction, multivariate logistic stepwise regression analysis (Table 2) showed that FDP (OR=1.047, 95% CI: 1.025–1.070, P < 0.001), TM (OR=1.215, 95% CI: 1.033–1.429, P = 0.019), and MA (OR=1.104, 95% CI: 1.026–1.188, P = 0.008) were all independent risk factors for VTE. This means that for each one-unit increase in the levels of these three indicators, the risk of VTE in patients increases by 4.7%, 21.5%, and 10.4% respectively. Moreover, this association is not affected by baseline factors such as gender, smoking history, and diabetes (Table 1 shows balanced baseline characteristics between the two groups). From the perspective of diagnostic efficacy, further analysis of the receiver operating characteristic (ROC) curve (Table 3) confirmed that the area under the curve (AUC) of the combined model of the three indicators reached 0.860 (95% CI: 0.789–0.931), which was significantly superior to that of individual indicators (AUC of FDP = 0.789, AUC of TM = 0.698, AUC of MA = 0.684). Meanwhile, its sensitivity (70.5%) and specificity (85.7%) achieved a clinically acceptable balance—it not only avoids the misdiagnosis risk caused by the excessively low specificity (55.4%) of TM alone, but also compensates for the missed diagnosis hidden danger due to the insufficient sensitivity (54.5%) of MA alone, providing a reliable quantitative basis for clinical risk stratification.

Notably, the selection of combined biomarkers in this study was not a random combination, but based on the core pathological mechanism of VTE occurrence: FDP reflects the degradation activity of fibrin (ogen) and serves as a direct marker for the activation of the coagulation-fibrinolytic system [51,52]; as a specific indicator of vascular endothelial injury [53,54], TM can early indicate the impairment of vascular wall integrity after trauma; MA, through thromboelastography (TEG), directly quantifies the maximum strength of blood clots and intuitively reflects the hypercoagulable state formed by the synergistic effect of platelets and fibrin [55–57]. These three markers respectively cover the key links of “endothelial injury-coagulation activation - thrombus formation”. Moreover, correlation analysis in this study (Fig 1) showed that there was no direct linear correlation between FDP, TM and TEG indicators (including MA), indicating that the three markers assess VTE risk from different dimensions. Their combined application can achieve “mechanistic complementarity”, which is also the core reason why their diagnostic efficacy is superior to that of a single indicator. In addition, this study strictly excluded patients with disseminated intravascular coagulation (DIC) (confirmed by clinical symptoms, coagulation parameters, FDP < 40 μg/mL and normal platelet count) [58,59]. This further clarifies that the combined model is suitable for VTE risk assessment in patients with simple traumatic fractures, avoiding the interference of confounding factors on the conclusions.

From the perspective of clinical practical application, this combined detection can be gradually integrated into the VTE risk diagnostic workflow for patients with traumatic fractures. The specific pathway is recommended as follows: Within 72 hours of patient admission (consistent with the sample collection time window of this study), simultaneously detect FDP, TM, and TEG (to obtain the MA value). For patients whose combined score of the three indicators (derived from the optimal threshold of the ROC curve, e.g., FDP > a certain value + TM > a certain value + MA > a certain value) reaches “high risk”, prioritize arranging CT or MRI to quickly confirm the presence of occult VTE (such as intra-abdominal venous thrombosis or asymptomatic DVT). For “moderate-risk” patients, combine with dynamic ultrasound follow-up (re-examination every 3–5 days) and strengthen anticoagulant prophylaxis. For “low-risk” patients, manage them according to the routine clinical pathway to reduce the burden of unnecessary imaging examinations. This workflow not only targetedly addresses the problem of high missed diagnosis rate of ultrasound, but also reduces the use of invasive examinations through biomarker pre-screening, which meets the clinical needs of “precision medicine” and “cost-effectiveness”. Especially for elderly patients with traumatic fractures (the mean age of the VTE group in this study was 66.52 years, which was significantly higher than 52.39 years of the control group, Table 1), this combined detection can identify high-risk populations earlier and provide a basis for timely intervention (such as adjusting anticoagulant regimens), thereby reducing the post-operative mortality related to VTE.

The results of this study also show good consistency and continuity with the conclusions of existing literature. The researches confirmed that the levels of D-dimer and FDP in trauma patients are closely related to the occurrence of VTE [60,61]. On this basis, this study further verified the independent predictive value of FDP, TM, and MA, and first confirmed that the combined diagnostic efficacy of the three is optimal, providing a new supplement to the existing VTE risk assessment system.

Several limitations of this study should be acknowledged. First, the retrospective single-center design and relatively small sample size (44 patients with VTE and 56 controls) may limit the generalizability of our findings. The limited sample size may also have contributed to instability in the observed event distribution and reduced the robustness of subgroup analyses. Therefore, larger multicenter prospective studies are warranted to validate our findings and to further explore potential subgroup differences across fracture locations, such as the lower extremities and spine. Second, although the combined use of FDP, TM, and MA demonstrated promising discriminatory performance, this study did not establish specific clinical cutoff values or a practical risk stratification framework for their combined application. Future studies should aim to develop and validate a multivariable prediction model or risk scoring system based on regression equations, in order to provide more clinically applicable thresholds for VTE risk assessment. Third, some potentially relevant coagulation-related variables, such as fibrinogen, were not included in the present analysis. Although FDP can reflect fibrin(ogen) metabolism to some extent [62], further studies are needed to determine whether additional biomarkers could improve model performance.

Another important limitation is the potential confounding effect of peri-admission hemostatic or anticoagulant therapy. Some trauma patients may receive individualized thromboprophylaxis before surgery, particularly when surgery is delayed, which could influence TEG parameters, especially MA. In addition, the lack of detailed fracture classification and injury-severity data limited our ability to fully account for trauma-specific heterogeneity. Different fracture patterns and hemorrhagic burden may independently affect FDP and TM levels through trauma-induced anemia, endothelial activation, and fibrinolytic changes. Therefore, residual confounding cannot be excluded. Finally, this study did not assess dynamic changes in FDP, TM, and MA over time. Future prospective studies with serial measurements may better clarify their temporal predictive value for VTE.

This combination not only covers the key pathological links of VTE occurrence, but also balances sensitivity and specificity. It can provide clinicians with a clear pathway “from laboratory indicators to clinical decision-making”—identifying high-risk patients through early screening, guiding the implementation of subsequent imaging examinations and preventive measures, and ultimately reducing the missed diagnosis rate and mortality of VTE in patients with traumatic fractures. Future studies should further explore this area, focusing on “quantitative thresholds” and “prospective validation,” to promote the integration of this biomarker combination into clinical routine diagnostic algorithms.

The coagulation indicators in trauma patients may fluctuate with their changing conditions over time. Future studies should monitor the coagulation indicators of trauma patients at multiple time points and conduct follow-up investigations on these patients after their discharge. Finally, the diagnosis in this study was mainly based on ultrasound results. However, ultrasound has its own leakage rate, which may cause an underestimation of the efficacy of the diagnostic model. This also implies that the actual diagnostic efficacy of this diagnostic model may be better than the model of the data derived from the study.

## Conclusion

In conclusion, the combined detection of FDP, TM, and MA shows promising potential for VTE risk stratification in post-traumatic fracture patients, but current evidence is insufficient for direct clinical implementation. The biomarkers reflect fibrin(ogen) turnover, endothelial injury, and clot strength for early risk assessment. However, its retrospective design, small sample size, and lack of longitudinal monitoring or genetic analysis limit generalizability. Future research should focus on multicenter prospective validation, dynamic biomarker tracking, cost-effectiveness studies, mechanistic explorations of biomarker interactions, and integrating genetic factors to refine models, enhancing clinical utility for personalized VTE prevention.

## Supporting information

S1 Data The data of the study of “Risk Prediction of Combined Fibrinogen Degradation Products, Thrombomodulin, and Maximum Amplitude for Assessing Association with Venous Thromboembolism in Patients with Post-Traumatic Fractures”. (XLSX)
